# A Case Report on the Open Surgical Repair of Thoracoabdominal Aortic Aneurysm With Severe Thoracic Vertebral Body Erosion. Experience in LMIC

**DOI:** 10.1002/ccr3.71115

**Published:** 2025-10-02

**Authors:** Natnael G. Admassu, Chernet T. Mengistie, Biruk T. Mengistie, Solyana Bereded, Yonas Nibret, Henok Teklesilase, Abebe Bezabh, Azaryas K. Admassu

**Affiliations:** ^1^ School of Medicine, College of Health Sciences, Addis Ababa University Addis Ababa Ethiopia; ^2^ Vascular Surgery Unit, School of Medicine, College of Health Sciences, Addis Ababa University Addis Ababa Ethiopia; ^3^ Cardiothoracic Surgery Unit, School of Medicine, College of Health Sciences, Addis Ababa University Addis Ababa Ethiopia; ^4^ Neurosurgery Unit, School of Medicine, College of Health Sciences, Addis Ababa University Addis Ababa Ethiopia

**Keywords:** case report, low‐resource setting, sagittal alignment, thoracoabdominal aortic aneurysm, vertebral erosion

## Abstract

Open surgical repair of thoracoabdominal aortic aneurysm with vertebral erosion is rare and complex, especially in low‐resource settings. This case shows that with multidisciplinary planning, staged aneurysm exclusion and spinal stabilization can yield excellent outcomes, even where endovascular tools are unavailable.

## 1 Introduction

Thoracoabdominal aortic aneurysms (TAAAs) involve progressive dilation of the descending thoracic aorta extending into the abdomen and remain among the most challenging vascular diseases [1]. Although TAAAs are uncommon, they carry very high morbidity and mortality if untreated [1, 2]. Because these aneurysms are often clinically silent until late, the true incidence is hard to measure and is usually extrapolated from surgical series rather than population screening [2]. Treatment has traditionally relied on open surgical repair, with endovascular and hybrid approaches emerging as adjuncts. Open repair allows direct exclusion of the aneurysm (often via thoracotomy and graft replacement), but it requires meticulous planning and multidisciplinary teamwork to manage the risks [3]. Previous studies emphasize that while endovascular tools are available, “the mainstay of TAAA treatment remains open surgical repair,” which demands careful patient evaluation and a collaborative surgical team [3].

Vertebral body erosion caused by aortic aneurysms is exceedingly rare [4, 5]. In general, vertebral lysis is far more often due to tumors, infection, or inflammation; aneurysms are an unusual culprit [6]. Only a few dozen cases have been documented in the English literature [7, 8, 9, 10, 11]. One study identified 42 reported cases of aortic aneurysm eroding the spine (mostly infrarenal AAAs) up to 2012 [5], and a recent systematic review tallied roughly 80 patients with vertebral erosion from chronic aneurysm rupture [6]. In these cases, the aneurysms tended to be large (mean ~7 cm) and chronic, and all patients had hypertension [6]. The proposed mechanism is chronic contained rupture or inflammation: a periaortic hematoma and constant pulsation slowly erode the anterior vertebral cortex [6, 12]. Clinically, this can present as chronic back pain and radicular symptoms, often mimicking disc disease and leading to delayed diagnosis [1, 5]. When aneurysm‐related vertebral erosion is identified, surgical intervention is generally indicated due to the risk of catastrophic rupture [3, 12].

Managing TAAAs is particularly difficult in low‐resource settings. Low‐income countries often lack endovascular capability and have very few vascular specialists: for instance, Ethiopia has only about 0.25 vascular surgeons per 10 million population (vs. ~100/10 M in the U.S.) [13]. Despite these constraints, Innovative solutions exist [14]. For example, a 10‐year Japanese series using hybrid visceral debranching plus TEVAR showed promising results [15], but such approaches require advanced equipment and expertise. In many places without these resources, open repair remains the only feasible option [3]. Reports of combined vascular and spine procedures for aneurysm‐induced osteolysis are very scarce, especially from low‐ and middle‐income countries. Here we describe a 63‐year‐old Ethiopian woman with a large TAAA causing severe thoracic vertebral collapse and kyphosis, successfully managed by staged open aneurysm repair followed by posterior spine stabilization. This case highlights that even in a low‐resource context, a carefully planned multidisciplinary approach can treat this rare and dangerous condition.

## 2 Clinical History/Examination

A 63‐year‐old Ethiopian woman with 5 years of poorly controlled hypertension presented with 3 months of progressive, severe mid‐back pain radiating to the legs with tingling. She had become essentially unable to sit or stand upright due to the pain and resultant spinal deformity. On exam, she was hemodynamically stable but sat in a markedly hunched (kyphotic) posture. Motor and sensory function were intact in all limbs, but straightening her spine was impossible due to pain. Routine labs (CBC, renal function, ESR, CRP) were normal. Echocardiography showed a calcified aortic valve with mild AR and Grade II diastolic dysfunction with a maintained LVEF of 65%.

## 3 Differential Diagnosis, Investigations, and Treatment

**FIGURE 1:**
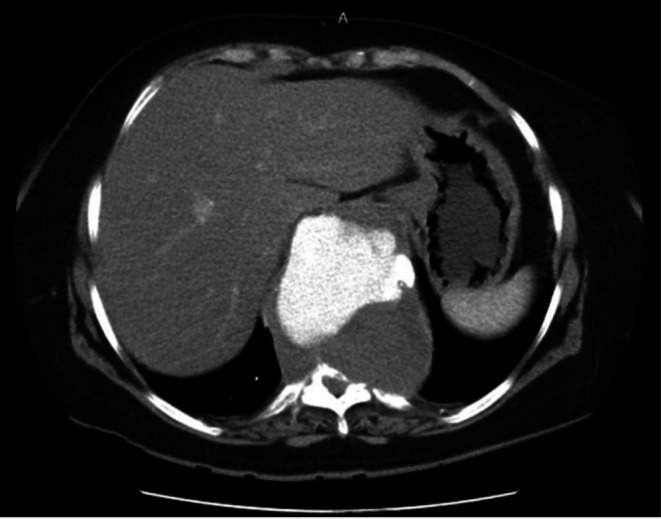
Preoperative axial CT angiography showing a large thoracoabdominal aortic aneurysm compressing adjacent thoracic structures. The aneurysm sac is visible at its maximum diameter (~7.0 cm), with early signs of surrounding vertebral body remodeling.

**FIGURE 2:**
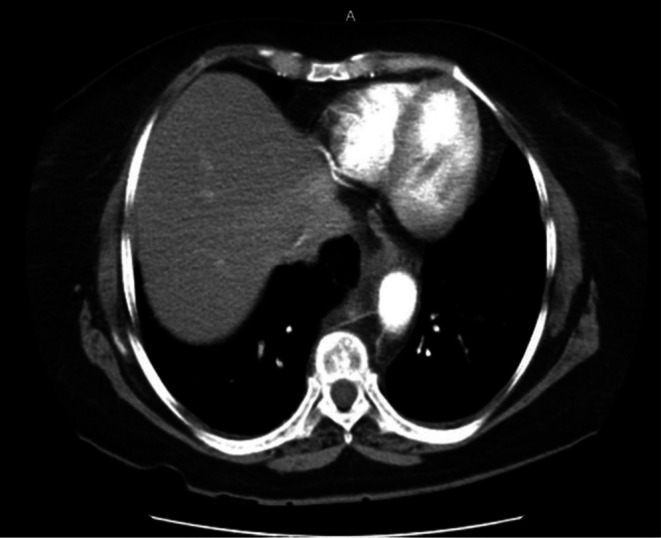
Preoperative coronal CT angiography showing extension of the aneurysm along the thoracic and abdominal aorta. The image illustrates the close proximity of the aneurysm to the vertebral column and adjacent structures.

**FIGURE 3:**
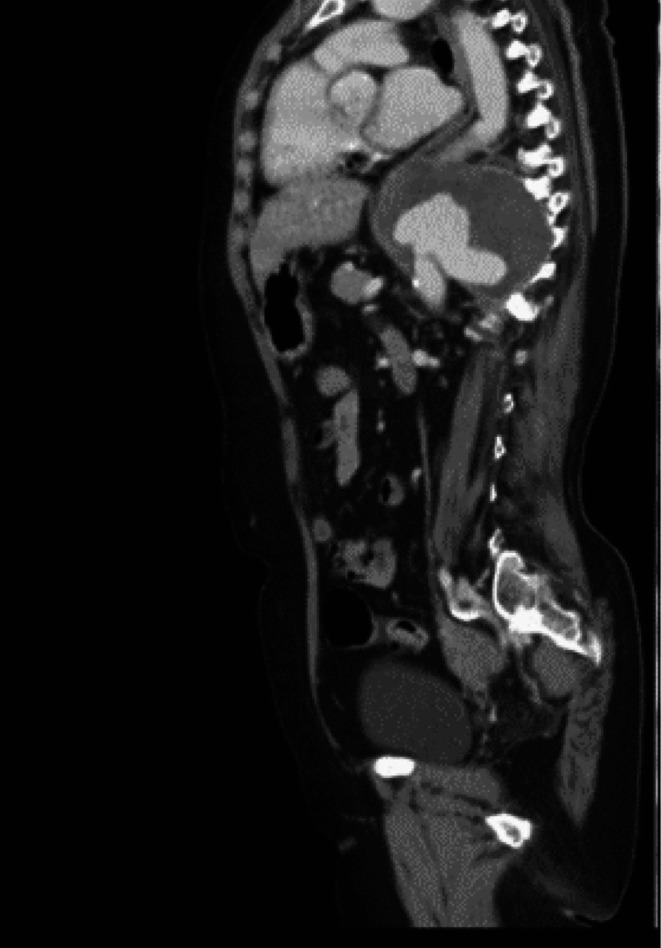
Preoperative sagittal CT reconstruction demonstrating near‐complete lytic erosion and collapse of the T9 to T11 vertebral bodies. This erosion results in a focal kyphotic deformity and anterior angulation of the spine.

**FIGURE 4:**
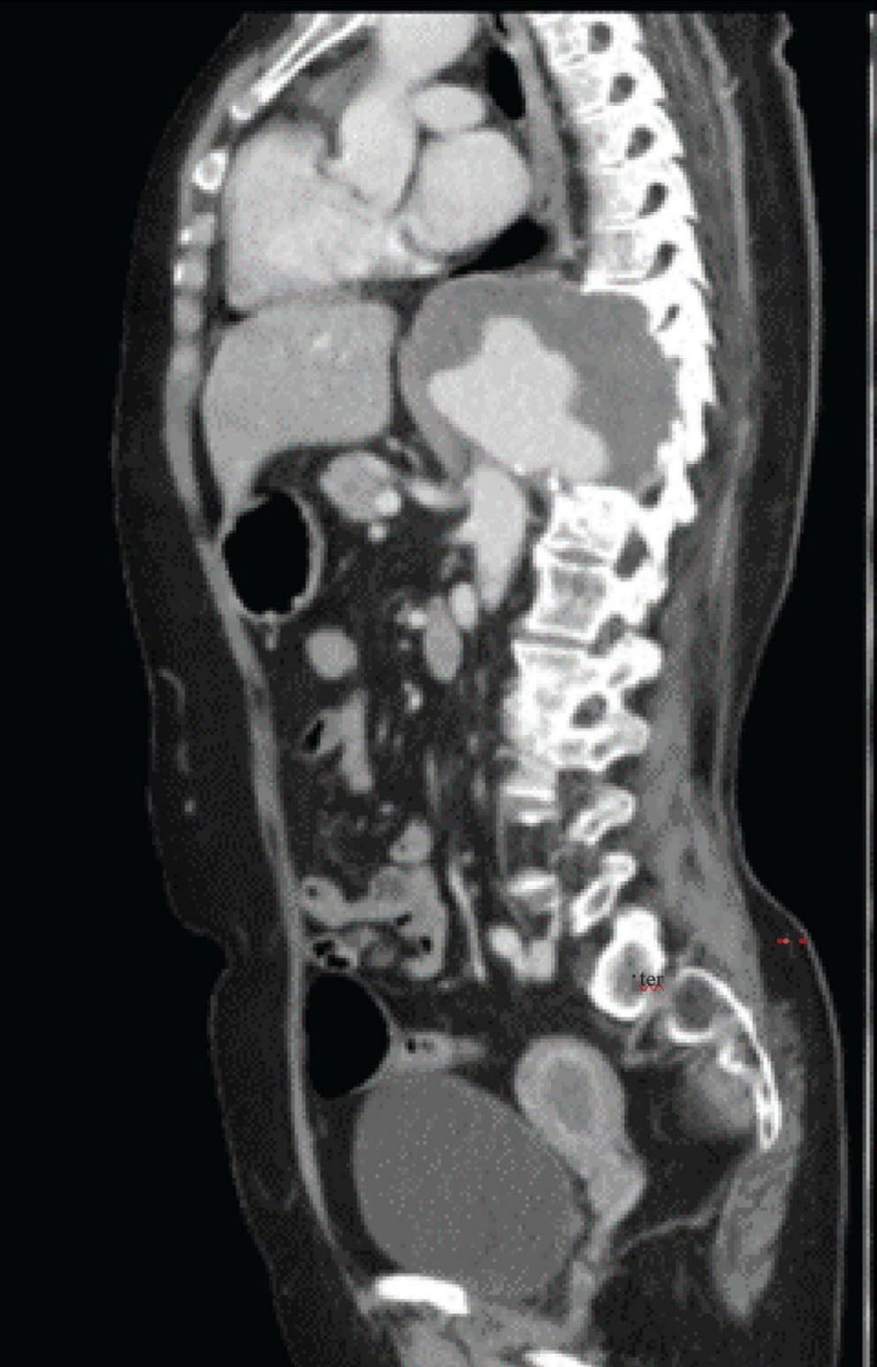
Sagittal CT close‐up view focusing on the thoracic vertebrae. T9 to T11 are markedly collapsed, with partial involvement of T8. The anterior vertebral cortex is eroded, indicating pressure effects from chronic aneurysmal expansion.

**FIGURE 5:**
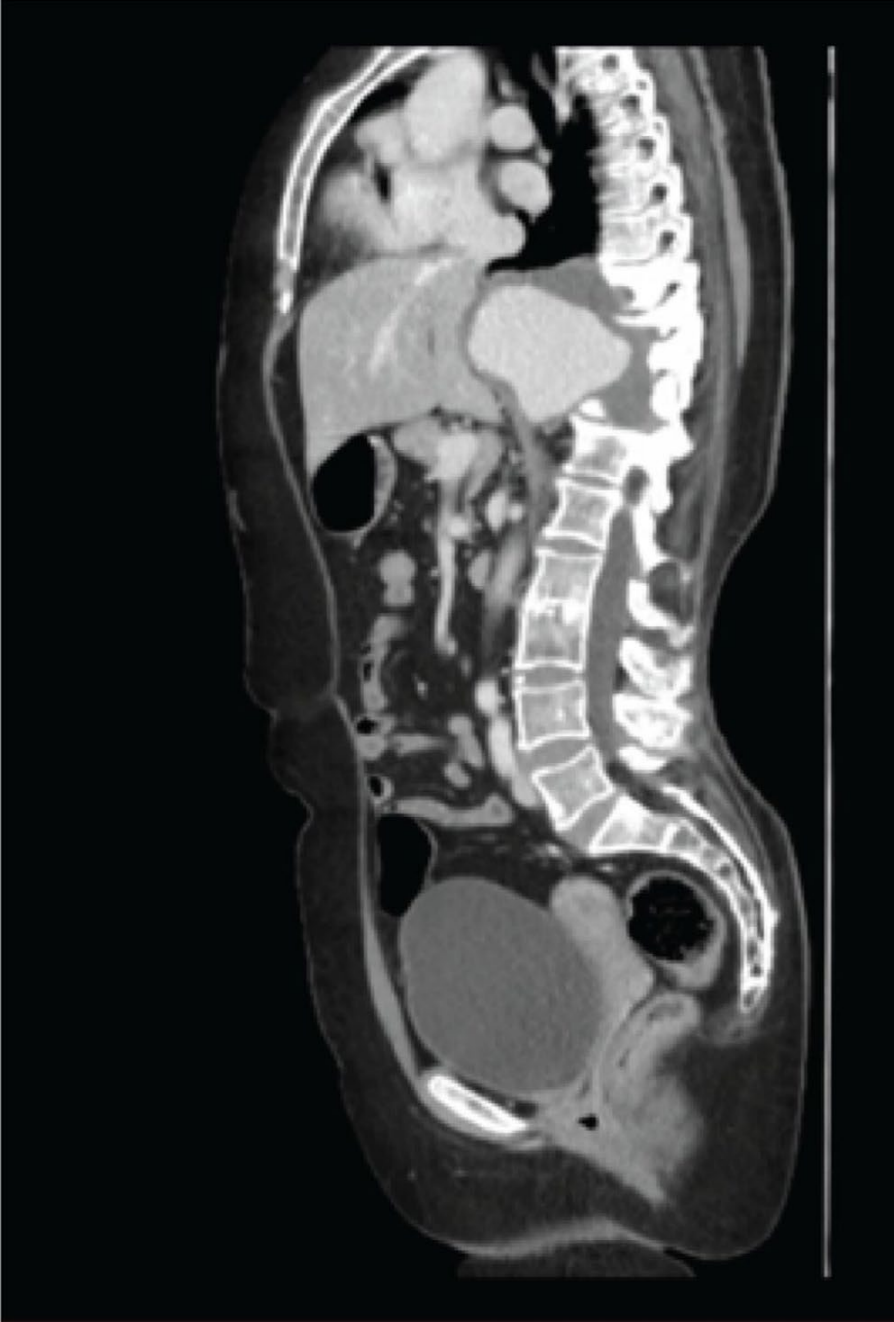
3D CT reconstruction of the thoracolumbar spine and aorta showing severe kyphotic deformity due to vertebral collapse. The spatial relationship between the aneurysm and vertebral elements is clearly visualized.

**FIGURE 6:**
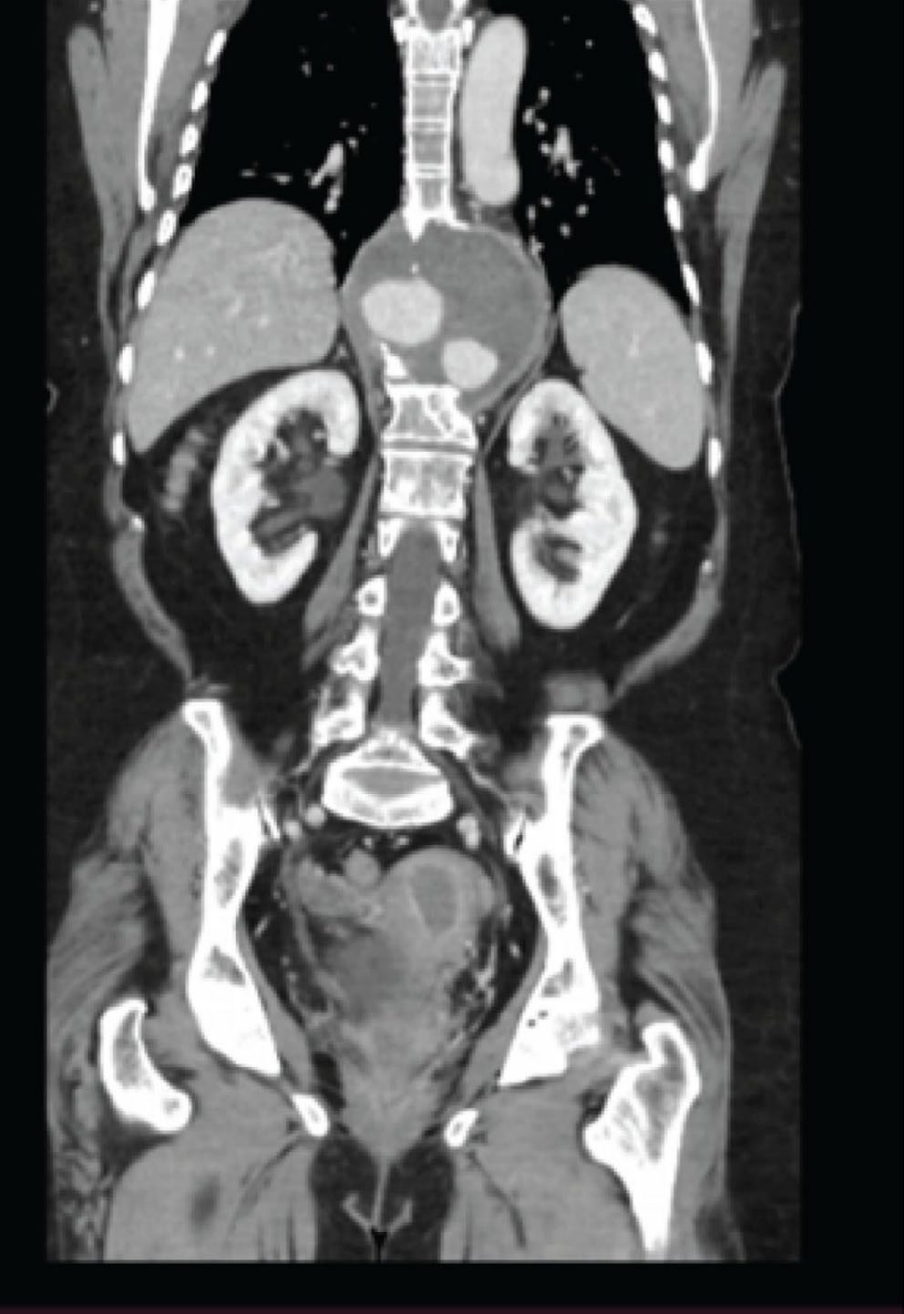
Coronal CT angiographic view showing asymmetrical involvement of the vertebral bodies. Erosion is more pronounced on the left side, correlating with the left‐sided origin of the aneurysm bulge.

CT angiography revealed a very large aneurysm of the descending thoracic and upper abdominal aorta, measuring about 7.0 cm at its widest point (Figures 1 and 2). Notably, the aneurysm had eroded the anterior portions of the T9–T11 vertebral bodies (and partially T8 on the left). Sagittal reconstructions showed near‐complete collapse of T9–T11 with a severe kyphotic angulation (estimated Cobb angle ~60°) (Figures 3, 4, 5, 6). The cervical plumb line was markedly anterior to the sacrum (> 5 cm), indicating gross sagittal imbalance. A dual‐energy X‐ray absorptiometry (DEXA) scan showed osteopenia (T‐score 1.8), which likely contributed to the vertebral collapse under the chronic aneurysmal pressure.

We elected a two‐staged open approach. This was because a single‐stage combined aortic replacement and multilevel spinal reconstruction would have required prolonged anesthesia, a very large transfusion, and conflicting surgical exposures (left thoracotomy vs. prone posterior), all of which posed unacceptably high perioperative risk given the patient's comorbidities and our limited ICU/blood bank resources. Prioritizing aneurysm repair addressed the immediate life threat (rupture risk) and removed the mechanical driver of vertebral destruction, thereby reducing intraoperative hemorrhage risk during the later vertebrectomy and permitting safer, better‐planned posterior instrumentation after recovery.

In multidisciplinary planning, the goals were: (1) completely exclude the aneurysm with a graft; (2) decompress any neural elements if needed; and (3) restore spinal alignment as much as possible (aiming for sagittal balance with C7 plumb line < 5 cm). Two months apart, we performed two operations. The 2‐month interval between procedures was selected to allow recovery from the high‐risk vascular operation, optimization of the patient's medical status (blood pressure control, nutrition, and anemia correction), confirmation of successful aneurysm exclusion on interval imaging, and careful planning of the spinal reconstruction.

**FIGURE 7:**
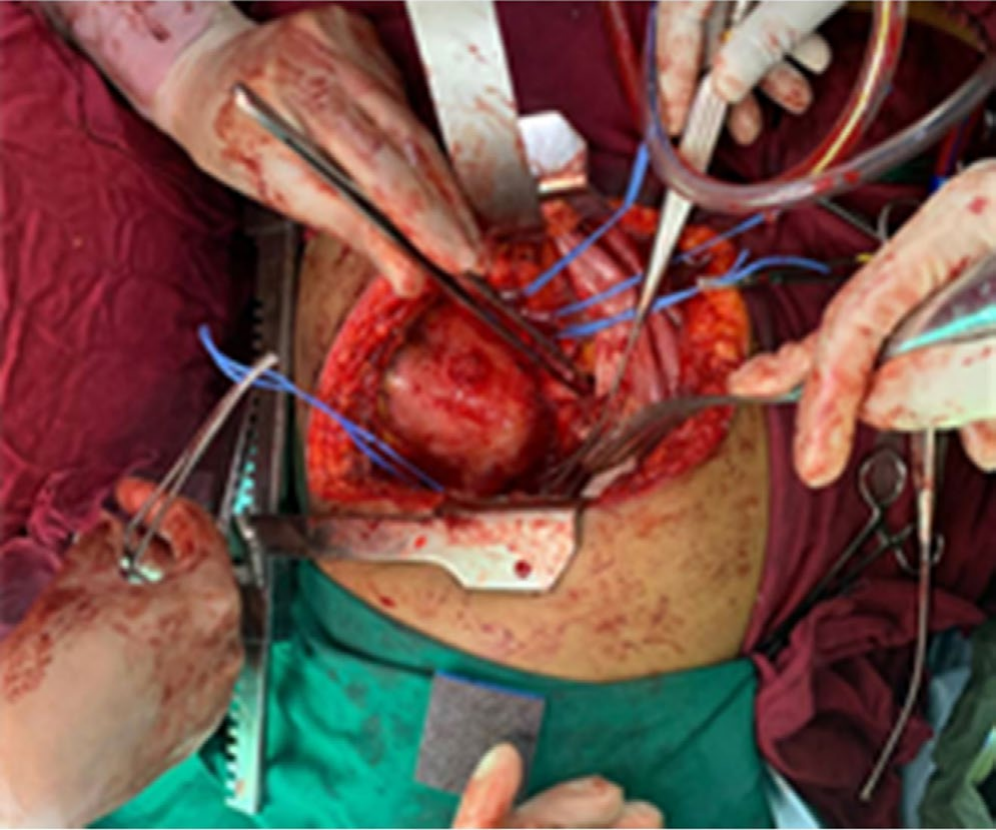
Intraoperative image during thoracotomy showing the exposed thoracoabdominal aortic aneurysm prior to excision. The aneurysm wall is thinned, and surrounding tissues are displaced.

**FIGURE 8:**
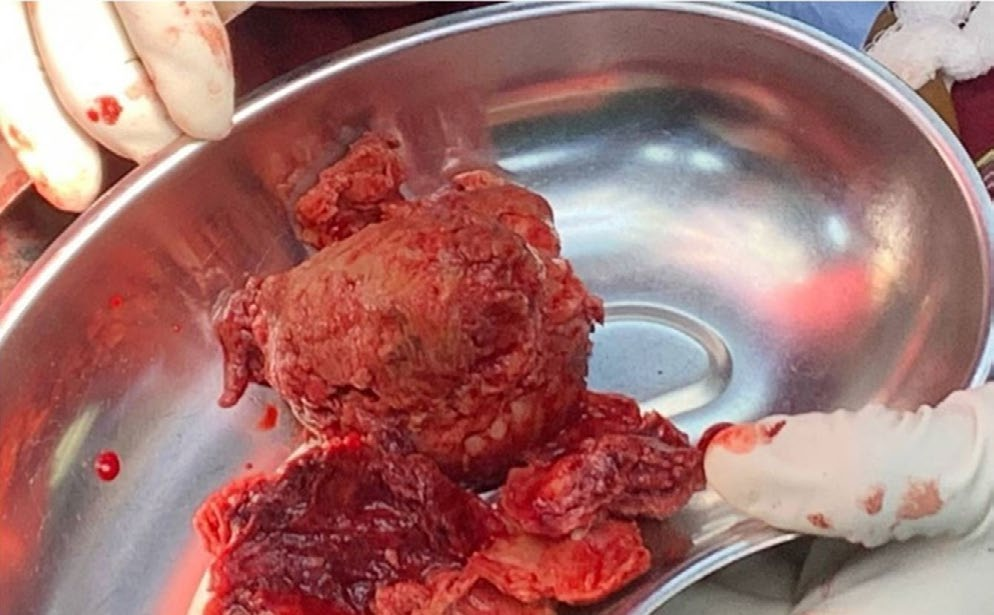
Intraoperative field after aneurysm excision, demonstrating the open aortic segment and preparation for graft placement. Temporary vascular control is established proximally and distally.

**FIGURE 9:**
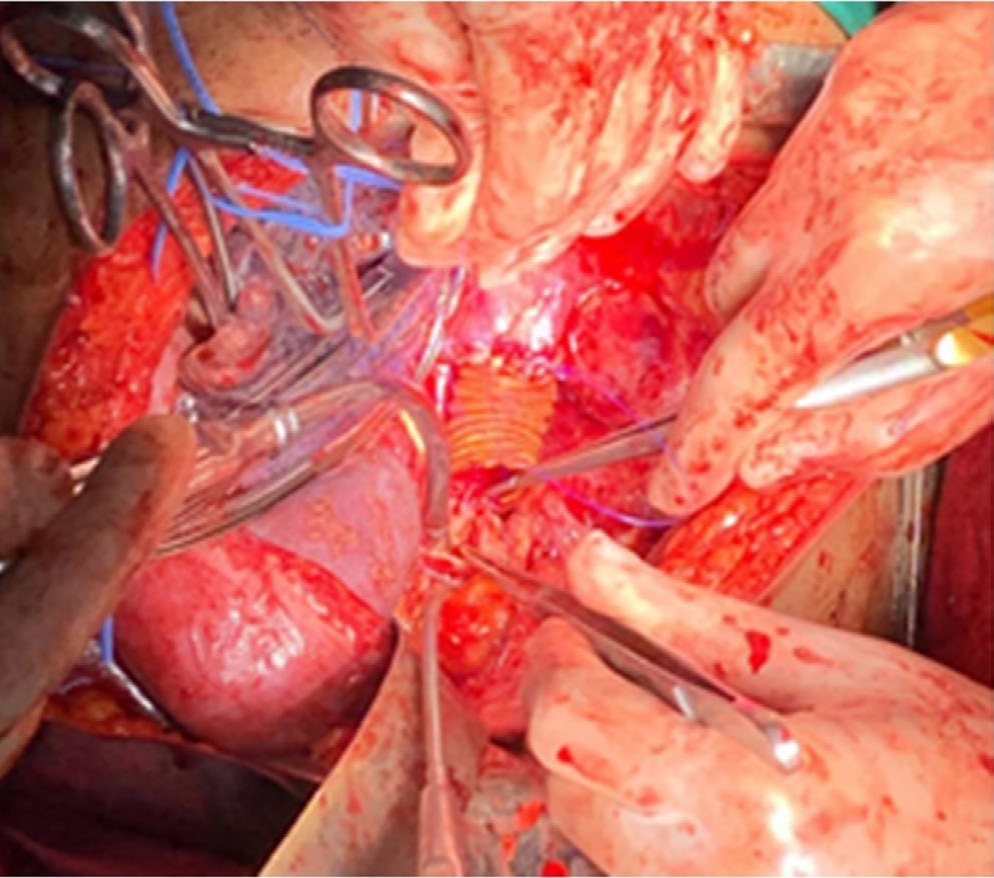
Placement of the Dacron graft replacing the excised aneurysmal segment. The graft is secured with proximal and distal anastomoses between healthy aortic ends above T8 and below T12.

First operation: A left posterolateral thoracotomy (via the 6th interspace) was performed. The diaphragm was partially divided (phrenotomy) to expose the aorta. The aneurysmal aorta from above T8 to below T12 was replaced with a 26‐mm Dacron graft. All intercostal branches feeding T9–T12 were ligated. Preoperative CTA did not clearly identify the artery of Adamkiewicz, and preservation of these branches was not feasible; spinal cord protection relied on intraoperative neuromonitoring (motor and somatosensory evoked potentials, which remained stable), strict avoidance of hypotension, prompt transfusion, and meticulous hemodynamic management. Estimated blood loss was ~2.5 L, managed with transfusion and cell salvage (Figures 7, 8, 9).

**FIGURE 10:**
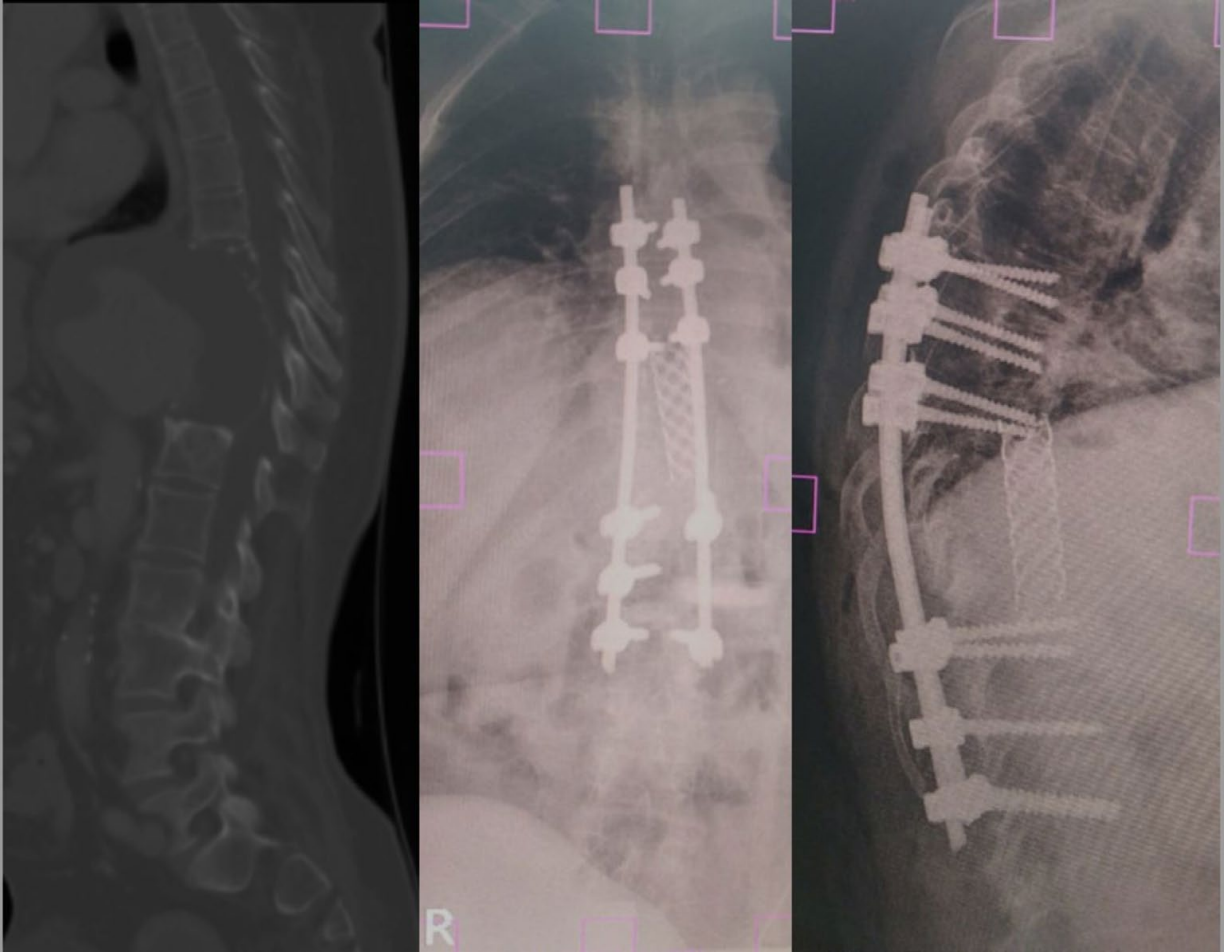
Spinal reconstruction images. (A) Preoperative sagittal CT showing severe anterior vertebral body erosion and kyphotic deformity centered at T9–T11. (B) Postoperative axial CT demonstrating central placement of the interbody cage within the resected vertebral space. (C) Postoperative sagittal CT or X‐ray showing correction of kyphosis and posterior instrumentation spanning T6 to L2 with pedicle screws and rods.

Second operation: A midline posterior thoracolumbar approach. A modified left costotransversectomy was performed at T9–T11. The grossly collapsed T9–T11 vertebrae were resected. A titanium expandable cage filled with iliac crest bone graft was inserted anteriorly to bridge the defect (Figure 10). Posterior pedicle screw instrumentation was placed from T6 to T8 and L1 to L2 (three levels above and below the defect), connected with rods, to achieve rigid fixation and reduce kyphosis.

## 4 Outcome and Follow‐Up

The postoperative courses were uneventful. After each stage, she was extubated on post‐op day 2 in the ICU. There were no postoperative complications related to the vertebrectomy or interbody cage during the documented follow‐up: no surgical site infection, no radiographic hardware failure, and progressive clinical improvement. The surgical aim for the spinal reconstruction was restoration of sagittal balance, aiming for a C7 plumb line ≤ 5 cm anterior to the sacrum, together with substantive reduction of the focal kyphosis. On the immediate postoperative lateral radiograph (Figure 10), the Cobb angle measured using the standard Cobb technique was approximately 0°, indicating near‐complete correction of the focal kyphotic deformity and restoration of sagittal balance. Following the spinal reconstruction, she began mobilizing with a brace within 1 week. By 3 months postoperatively, her back pain had diminished dramatically, and she could sit and stand with minimal support. The lower limb paresthesias resolved completely. At 6 months of follow‐up, she continued to demonstrate sustained functional improvement, remained pain‐free, and had no new neurological deficits. She remains under surveillance by the vascular and spine teams and reports satisfaction with her outcome.

## 5 Discussion

TAAAs require very complex management due to their size, extent, and involvement of major branches. As shown in major reviews, untreated TAAAs carry high mortality [2]. Although endovascular methods are increasingly used, open repair remains the standard of care where feasible [3]. Open TAAA repair (using Dacron graft replacement via thoracotomy) provides direct aneurysm exclusion and decompression of surrounding structures, but it demands careful preparation (e.g., planning the incision, spinal cord protection, managing intercostal reimplantation) [3, 14]. Our first‐stage thoracotomy followed classic open techniques, which literature supports as effective for definitive aneurysm management [3]. In resource‐limited environments without stent‐graft availability, this open approach may be the only option [16], and our case demonstrates it can be done safely when executed by an experienced team. Notably, in modern series, hybrid procedures can achieve low perioperative mortality, around 5% 30‐day mortality with hybrid TAAA repair [15], but such resources were not available here.

Systematic reviews and recent case series emphasize that vertebral body erosion from aortic aneurysms is extremely unusual but increasingly recognized, commonly presenting with back pain and often misdiagnosed as infection or malignancy, which makes high clinical suspicion and cross‐sectional vascular imaging essential for diagnosis [4, 6, 17]. Our case resembles the “chronic contained rupture” scenarios reported in prior series: a large aneurysm, longstanding hypertension, and a periaortic hematoma gradually eroding the vertebral bodies [6, 12]. These studies have emphasized that aneurysm‐related vertebral erosion is rare, comprising only a few percent of aneurysm cases [6, 12]. All documented cases had large aneurysms (mean > 7 cm) [6] and systemic hypertension, mirroring our patient's profile. The clinical lesson is that in any hypertensive patient with chronic back pain, one must consider vascular causes; otherwise, the diagnosis may be delayed [5, 12]. In our patient, the catastrophic risk (and debilitating pain) justified urgent surgery even though she had no neurologic deficits. Indeed, vertebral erosion can cause severe pain, and rupture risk mandates definitive repair [12].

Restoration of spinal stability and alignment is a critical adjunct in these cases. Reported strategies range from single‐stage repair to staged and hybrid approaches, but recent reports suggest staged open repair with delayed spinal reconstruction may lower physiologic stress and perioperative morbidity, especially in resource‐limited settings [18, 19, 20]. Once the aneurysm was excluded, we addressed the now‐collapsed thoracic spine with anatomic reconstruction. The use of an anterior cage and long‐segment pedicle screw fixation to correct kyphosis is well established in spinal reconstruction. By spanning multiple levels above and below the defect, we achieved solid support. Neuromonitoring confirmed safety during correction. When vascular and spine surgeons collaborate, combined staged operations can be performed without excess risk and with good outcomes [3, 16]. Our outcome, marked pain relief, return of upright posture, and preserved neurologic function, is consistent with that experience.

Finally, the context of our low‐resource setting merits emphasis. With only a quarter of a vascular surgeon per 10 million people in Ethiopia [13], undertaking such a complex case is exceptional. Yet this case shows that with thorough preoperative planning and teamwork, even the most challenging aneurysm pathologies can be managed outside high‐income centers. Quantifying the spine deformity (kyphotic angle, sagittal balance) and targeting realignment were key steps in our planning. In summary, a thoracoabdominal aneurysm with adjacent vertebral erosion is a rare, life‐threatening condition. Our experience suggests that an open, staged repair can be successful even in resource‐limited environments [3, 12]. We encourage reporting of similar cases and collaboration between specialties to improve awareness and outcomes for this formidable problem [3, 6].

## 6 Conclusion

Thoracoabdominal aortic aneurysm with secondary vertebral body erosion is an exceptionally rare and serious condition. Our experience shows that even in a low‐resource setting, successful management is possible through thorough preoperative assessment, multidisciplinary planning, and staged surgery. Key elements include defining the extent of spinal deformity (quantitatively) and aiming for sagittal realignment, in addition to securing aneurysm repair. This case adds to the limited literature on combined vascular–spine interventions for aneurysm‐induced osteolysis and underscores that open surgical repair remains the mainstay treatment when endovascular options are unavailable. Further collaboration and case reporting from diverse settings will improve understanding and outcomes for this challenging problem.

## Author Contributions

**Natnael G. Admassu:** conceptualization, writing – original draft. **Chernet T. Mengistie:** data curation, resources, writing – original draft. **Biruk T. Mengistie:** visualization, writing – original draft, writing – review and editing. **Solyana Bereded:** visualization, writing – review and editing. **Yonas Nibret:** conceptualization, writing – original draft. **Henok Teklesilase:** resources, supervision. **Abebe Bezabh:** supervision. **Azaryas K. Admassu:** resources, supervision.

## Ethics Statement

IRB review and approval was waived for this case report.

## Consent

Written informed consent was obtained from the patient for publication of the case details and accompanying images.

## Conflicts of Interest

The authors declare no conflicts of interest.

## Data Availability

The data that support the findings of this study are available from the corresponding author upon reasonable request.
